# Use of *in vitro* assays to assess the potential antiproliferative and cytotoxic effects of saffron (*Crocus sativus* L.) in human lung cancer cell line

**DOI:** 10.4103/0973-1296.71799

**Published:** 2010

**Authors:** Saeed Samarghandian, Mohammad Hossein Boskabady, Saideh Davoodi

**Affiliations:** *Department of Physiology, School of Medicine, Mashhad University Medical Sciences, Mashhad, Iran*

**Keywords:** Cytotoxicity, L929, lung cancer, MTT, saffron

## Abstract

**Background:**

Saffron is harvested from the dried, dark red stigmas of *Crocus sativus* flowers. It is used as a spice for flavoring and coloring food as a perfume. It is often used for treating several diseases. We investigated the potential of the ethanolic extract of saffron to induce antiproliferative and cytotoxic effects in cultured carcinomic human alveolar basal epithelial cells in comparison with non-malignant (L929) cells.

**Materials and Methods:**

Both cells were cultured in Dulbecco’s modified Eagle’s medium and treated with the ethanolic extract of saffron at various concentrations for two consecutive days. Our study resulted in sequences of events marked by apoptosis, such as loss of cell viability, morphology changes that were evaluated by MTT assay and invert-microscope, respectively.

**Results:**

The results showed that the ethanolic extract of saffron decreased cell viability in malignant cells as a concentration and time-dependent manner. The IC _50_ values against the lung cancer cell line were determined as 1500 and 565 μg/ml after 24 and 48 h, respectively. However, the extract at different concentrations could not significantly decrease the cell viability in L929 cells. Morphology of MCF7 cells treated with the ethanolic extract confirmed the MTT results.

**Conclusion:**

We also showed that even higher concentrations of saffron is safe for L929, but the extract exerts pro-apoptotic effects in a lung cancer-derived cell line and could be considered as a potential chemotherapeutic agent in lung cancer.

## INTRODUCTION

Lung cancer is the most frequent cause of cancer-related death and accounts for more than a million deaths yearly worldwide, with non-small cell lung cancer accounting for 75–85% of lung cancer.[1] Lung cancer is the second most common cancer in men after liver cancer. Cancer therapy is generally classified into three categories: surgery, radiation therapy and chemotherapy. Chemotherapy is the administration of drugs that can regulate the uncontrolled proliferation of abnormal cancer cells. The majority of chemotherapeutic drugs can be divided into alkylating agents, antimetabolites and anthracycline.[2] Although the use of molecular targeting drugs such as the tyrosine kinase activator imatinib is increasing, there are few drugs that achieve a complete recovery in cancer patients, and the failure of conventional chemotherapy to effect a major reduction in mortality indicates that the development of more effective chemotherapeutic drugs is essential for the treatment of cancer worldwide. In experimental cancer chemotherapy studies, attempts are made to identify agents that can exhibit any or a combination of the following characteristics: (i) prevent the initiation of tumors, (ii) delay or arrest the development of tumors, (iii) extend the cancer latency period, (iv) reduce cancer metastasis and mortality and (v) prevent recurrence of second tumors. The major focus of research in chemotherapy for cancer in recent times includes the identification, characterization and development of new and safe cancer chemopreventive agents.[3]

There has been growing interest in the use of naturally occurring compounds with chemopreventive and chemotherapeutic properties in the treatment of cancers. Herbs have been considered natural and valuable sources for anticancer drug discovery. Herbal medicine has been prescribed in many countries over centuries for treating various diseases, including infectious and malignant diseases. Plants have played an important role as a source of effective anticancer agents, and it is significant that 60% of the currently used anticancer agents are derived from natural sources including plants, marine organisms and microorganisms such as Taxol, a natural product isolated initially from *Taxus brevifolia* (Pacific Yew).[4] Saffron, the dry stigmas of the plant *Crocus sativus* L., belongs to the Iridaceae family and is cultivated in Iran and Spain.[5,6] The use of saffron dates back to ancient Egypt and Rome, where it was used as a dye in perfume and as a spice for culinary purposes. Although it is currently used as a spice and food colorant, however, traditional medicines have used saffron in the treatment of numerous illnesses, including cough, colic, insomnia, chronic uterine hemorrhage, cardiovascular disorders and tumors.[7–10] In the recent past, saffron is candidate for its anticancer and antitumor properties and, specially, its cytotoxic effect has been studied in the breast cancer cell lines, MCF-7.[11] However, there is no evidence on the therapeutic effects of saffron in the lung cancer cell line. Therefore, the aim of the present study was to assess the potential cytotoxic and antiproliferative effects of saffron (*C. sativus* L.) in human lung cancer cell lines.

## MATERIALS AND METHODS

### Material

3-(4, 5-Dimethylthiazol-2-yl)-2, 5-diphenyl (MTT) was purchased from Bioseen Technology Inc. (Shanghai, China) Dulbecco’s modified Eagle’s medium (DMEM) was purchased from Gibco BRL (Grand Island, NY, USA). Saffron was purchased from Saharkhiz Saffron Co. (SSC) (Mashhad, Iran) and fetal bovine serum was purchased from PAA Laboratories GmbH, Austria. Other chemicals were of the highest, commercially available quality.

### Preparation of the saffron extract

Saffron was supplied by Saharkhiz Saffron Co. and was processed in the Pharmacological Research Centre of Medicinal Plants. The part of *C. sativus* that is being used as additive and also as herbal medicine is the stigma. The stigma part of saffron was air dried in the shade before extraction. After grinding, a 1 g weight of the dried stigma was extracted with 10 ml ethanol (96%) for 2 h in an ultrasonic bath. The extract was filtered and concentrated in a vacuum evaporator. Then, the extract was kept at 2–6°C (refrigerator). The yield of extraction was around 35%.

### Cell culture conditions

The human non-small lung cancer cells (A549) and normal fibroblast mouse (L929) cell (as control) were obtained from Pasteur Institute (Tehran, Iran). Cells were maintained at 37°C in a humidified atmosphere (90%) containing 5% CO _2_ and subcultured every 3–4 days. Malignant and non-malignant cells were cultured in DMEM with 5% (v/v) fetal bovine serum, 100 units/ml penicillin and 100 μg/ml streptomycin.

### MTT colorimetric assay

The cell viability was determined using a modified 3-(4, 5-dimethylthiazol-2-yl)-2, 5-diphenyl tetrazolium (MTT) assay.[12,13] Briefly, cells were seeded (1 × 10^3^ cells/well) onto flat-bottomed 96-well culture plates. Saffron, at different concentrations (500, 1000, 1500 and 2000 μg/ml), was added to the wells and allowed to grow for 24 and 48 h. For each concentration and time course study, there was a control sample that remained untreated and received an equal volume of medium. After removing the medium, cells were then labeled with MTT solution (5 mg/ml in PBS) for 4 h and the resulting formazan was solubilized with DMSO (100 μl). Absorbance was measured at 550 nm using an automated microplate reader (Bio-Rad 550, Illinois, USA). Cell viability was expressed as a percentage of the control culture value. Experiments for each extract were carried out in triplicate, including untreated cell control and a blank cell-free control. The cytotoxic effects of the saffron extract on the lung cancer cell line was expressed as the IC _50_ value (the drug concentration reducing the absorbance of treated cells by 50% with respect to untreated cells).

### Morphologic analysis using an inverted microscope

Morphological studies using a normal inverted microscope were carried out to observe the morphological changes of cell death in malignant and non-malignant cell lines elicited by the ethanolic extract of saffron. Concentrations of 500 and 1500 μg/ml of saffron extracts were used for the morphological studies. The untreated cells served as the negative control. The morphological changes of the cells were visualized under the normal inverted microscope after 24 and 48 h post-treatment.

### Statistical analysis

All results were expressed as mean ± SEM. The significance of difference was evaluated with ANOVA and Bonfrroni’s test. A probability level of *P* <0.05 was considered statistically significant.

## RESULTS

To discriminate between the early and late effects of saffron action, malignant (A549) and non-malignant control (L929) cells were exposed to increasing concentrations of saffron for 24 and 48 h.

### Effect of ethanolic extract of saffron on cell viability

#### Morphological evaluation

**Figure 1:**
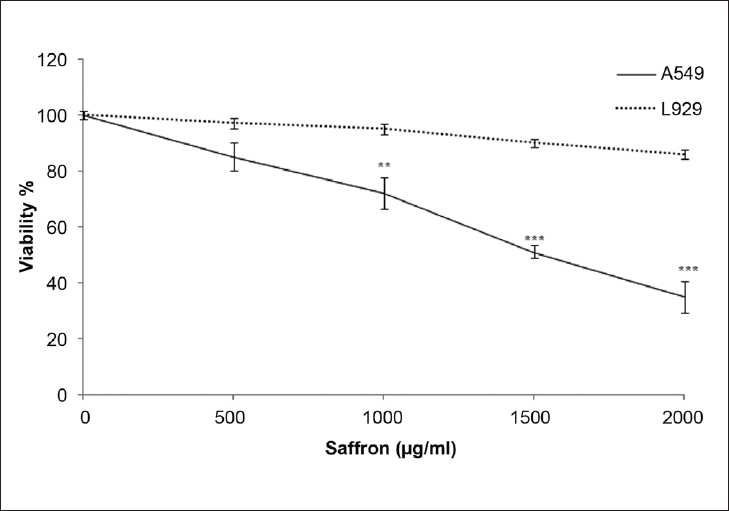
Comparison of cytotoxic effects of ethanolic extract of saffron on lung cancer cell (A549) and non-malignant cell (L929) line. Cells were treated with different concentration of saffron extract for 24 hours. Viability was quantitated by MTT assay. Results are mean ± SEM (n=6). The asterisks are indicator of statistically difference obtained separately at different time points compared to their controls shown in figure as **P <0.01, ***P <0.001

**Figure 2:**
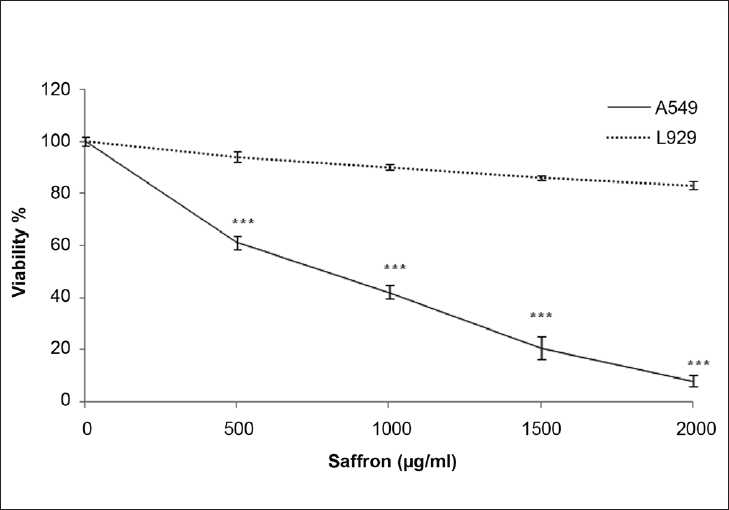
Comparison of cytotoxic effects of ethanolic extract of saffron on lung cancer cell (A549) and non-malignant cell (L929) line. Cells were treated with different concentration of saffron extract for 48 hours. Viability was quantitated by MTT assay. Results are mean ± SEM (n=6). The asterisks are indicator of statistically difference obtained separately at different time points compared to their controls shown in figure as ***P <0.001

After 24 h of incubation with the ethanolic extract of saffron (500 and 1500 μg/ml), morphologic changes were observed in the lung cancer cells versus L929 cells, which consisted of reduction in number of living cells, volume and rounding until the nucleus constituted the majority of the cellular volume. The reduction of malignant compared with non-malignant cells was statistically highly significant. This cytotoxicity was increased at higher concentrations [Figure 1]. After 48 h of incubation with saffron, morphological changes were observed in the A549 cell line even at the lowest dose (500 μg/ml). Thus, saffron-treated lung cancer cells (500 μg/ml) showed damage in the malignant cells, but there were no morphological changes in the saffron-treated L929 cells at the same concentration, this effect again becoming obvious at higher concentrations (1500 μg/ml) [Figure 2]. After 24 and 48 h, no clear morphological changes were detected in the L929 cells at any dose of saffron [Figures 1 and 2].

### Effect of saffron on cell viability

**Figure 3:**
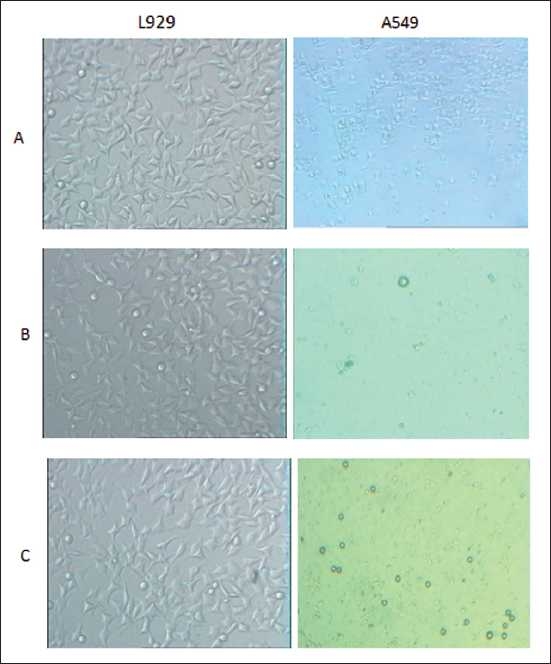
Comparison cytotoxicty effect of saffron extract on cell viability of lung cancer cell (A549) and non-malignant cell (L929) line. Morphological changes of cells after treatment with different concentration of saffron extract for 24 hours. A=control; B=500 (μg/ml); C= 1500 (μg/ml) saffron extract

**Figure 4:**
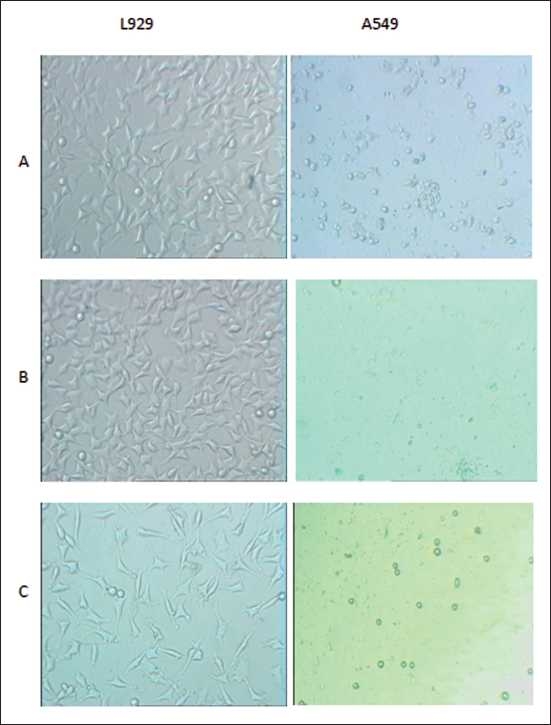
Comparison cytotoxicty effect of saffron extract on cell viability of lung cancer cell (A549) and non-malignant cell (L929) line. Morphological changes of cells after treatment with different concentration of saffron extract for 48 hours. A=control; B=500 (μg/ml); C= 1500 (μg/ml) saffron extract

In order to evaluate the effect of the ethanolic extract of saffron on the growth of human lung cancer cells and L929, the cells were incubated with different concentrations of the ethanolic saffron extract (500, 1000, 1500 and 2000 μg/ml) for 24 and 48 h, and their growth inhibitory effects were compared. The impact of the saffron extract on cell viability was quantitated by the MTT assay. The ethanolic saffron extract showed significantly high growth inhibitory effects on the lung cancer cell line in a concentration and time-dependent manner compared with the L929 cell line. As shown in Figure 3, the ethanolic extract of saffron (1000, 1500, 2000 μg/ml) decreased the cell viability in malignant cells but not in non-malignant cells after 24 h. This toxicity was consistent with morphologic changes. However, the extract, at different concentrations, could not significantly decrease the cell viability in L929 cells. After 48 h, a lower concentration of the ethanolic extract of saffron (500 μg/ml) dramatically decreased cell viability in the lung cancer cell line so that significant growth inhibition was initiated at 500 μg/ml after 48 h [Figure 4]. Therefore, exposure of the lung cancer cell line for 24 h significantly decreased the number of cells at a dose of 1000 μg/ml (*P* < 0.01), 1500 and 2000 μg/ml (*P* < 0.001). The dose-inducing 50% cell growth inhibition (IC_50_) against malignant cells was determined at 1500 and 565 μg/ml after 24 and 48 h, respectively [Table 1].

**Table 1:** Doses inducing 50% cell growth inhibition (IC_50_) of ethanolic saffron extract against lung cancer cell line (A549)

IC_50_	24h	48h
A549	1500(μg/ml)	565(μg/ml)

Cells were treated with different concentration of saffron extract for 24 and 48 hours. Viability was quantitated by MTT assay.

## DISCUSSION

Cancer is a growing health problem around the world. Natural products have long been used to prevent and treat many diseases, including cancer, and thus they are good candidates for the development of anticancer drugs.[14] In the present study, the cytotoxic and antiproliferative effects of the ethanolic saffron extract in carcinomic human alveolar basal epithelial cell lines, which, to our knowledge, are the first report on saffron-induced cytotoxicity in these cells, were investigated. Our results indicate that the saffron extract had a dose-dependent inhibitory effect on the growth of the human lung cancer cell line *in vitro*, but had no effect on the normal human cells, which is consistent with previous studies, indicating that saffron and its ingredients possess antitumor and anticarcinogenic activities.[15]

In the present study, saffron-induced cytotoxicity in the lung cancer cells was involved in the induction of morphological changes. The morphological features observed using the normal inverted microscope showed characteristic rounding of dying cells on treatment with saffron for 24 and 48 h compared with untreated controls. A number of *in vivo* and *in vitro* experiments indicate that saffron and its main ingredients have the potential to reduce the risk of developing several types of cancer. The saffron plant has been shown to be a source of bioactive compounds with cytotoxic, antitumoural, chemopreventive, antimutagenic and immuno-stimulating properties. Crocins, the major carotenoid components of saffron stigma, demonstrated antitumor properties, promoting tumor growth inhibition and increasing the life-span of treated tumor-bearing animals. Crocins are well tolerated and present no or minor side-effects. These, together with their water-solubility, make them suitable for chemotherapeutic use. Crocins and crocetins (the deglycosylated forms) were also found to be potent inhibitors of carcinogenesis as well as attenuators of the toxicity of some anticancer agents.[16] Crocins inhibit skin tumor promotion in mice (i.e., with benzo(a)pyrene). They have an inhibitory effect on the intracellular nucleic acid and protein synthesis in malignant cells as well as on protein kinase C (PKC) and prorooncogene in INNIH/3T3 cells, which is most likely due to their antioxidant activity.[17,18]

The interest on carotenoids as potential biomedical drugs is significantly growing. Carotenoids exhibit biological activities as antioxidants, affect cell growth regulation and modulate gene expression and immune response.[19,20] Several studies have demonstrated the use of some of them, such as β-carotene, α-carotene, lycopene, zeaxanthin or canthaxantine, in cancer prevention and therapy.[21,22]

Although the antioxidant and free radical scavenger properties of saffron and its ingredient (crocin) have been shown in previous studies,[23,24] however, carotenoids at high concentrations may act as pro-oxidants in biological systems.^20^ Therefore, it seems likely that potential compounds responsible for the inhibitory effect of saffron on tumor cell growth are its carotenoid ingredients. With respect to the mechanism(s) that may be involved, the intracellular level of sulfhydril (SH) compounds in tumor cells may be important factors partaking in the relative sensitivity of malignant cells to the effect of saffron[25] because it has been shown that the pre-treatment of tumor cells with saffron resulted in a doubling of the intracellular SH- compound levels. Thus, these results reveal that the saffron extract is non-toxic and that it possesses cytotoxic activity against the human lung cancer cell line. Several mechanisms attempting to explain the antitumor action at the cellular and molecular levels of the carotenoids present in saffron have been suggested:

- Modulation of programmed cell death, selectively promoting apoptosis in tumoural cells and inhibiting both internal and external apoptosis stimuli in non-tumoural cells.[26,27]
- Inhibition of cellular DNA and RNA synthesis, but not protein synthesis.
  Disruption of DNA–protein interactions has been proposed to explain this inhibition of nucleic acid synthesis.[28]
- Antioxidant activity, inhibition of free-radical chain reactions that could lead to oxidative damage and DNA alterations.[29]
- Enzymatic changes (GST, PKC), decreases in the formation of B (a) P adduct and reduction in the expression of proto-oncogenes.[30]

Studies on the cytotoxicity of carotenoids present in saffron produced controversial results concerning the comparative effects of glycosydic- and sugar-free carotenoids, but revealed that malignant cells are more sensitive than normal cells to the toxic effect of these compounds.[31] Our data also showed that the saffron extract has a higher cytotoxic activity against lung cancer cell lines than against non-malignant cells.

In conclusion, the present study supports increasing evidence that naturally occurring saffron extract may have an important role in cancer chemoprevention.

Taken together, the present study is the first to show the toxicity of saffron in the lung cancer cell lines. It could provide further knowledge to mechanisms involved in this toxicity. Saffron could also be considered as a promising chemotherapeutic agent in cancer treatment.
